# HMMEditor: a visual editing tool for profile hidden Markov model

**DOI:** 10.1186/1471-2164-9-S1-S8

**Published:** 2008-03-20

**Authors:** Jianyong Dai, Jianlin Cheng

**Affiliations:** 1 School of Electrical Engineering and Computer Science, University of Central Florida, Orland, FL 32816, USA; 2 Department of Computer Science, Informatics Institute, University of Missouri Columbia, Columbia, MO 65211, USA

## Abstract

**Background:**

Profile Hidden Markov Model (HMM) is a powerful statistical model to represent a family of DNA, RNA, and protein sequences. Profile HMM has been widely used in bioinformatics research such as sequence alignment, gene structure prediction, motif identification, protein structure prediction, and biological database search. However, few comprehensive, visual editing tools for profile HMM are publicly available.

**Results:**

We develop a visual editor for profile Hidden Markov Models (HMMEditor). HMMEditor can visualize the profile HMM architecture, transition probabilities, and emission probabilities. Moreover, it provides functions to edit and save HMM and parameters. Furthermore, HMMEditor allows users to align a sequence against the profile HMM and to visualize the corresponding Viterbi path.

**Conclusion:**

HMMEditor provides a set of unique functions to visualize and edit a profile HMM. It is a useful tool for biological sequence analysis and modeling. Both HMMEditor software and web service are freely available.

## Background

Hidden Markov Model (HMM) is a widely used statistical model for biological sequence analysis [1-6]. It has been used in many bioinformatics areas such as motif identification [5,6], gene structure prediction [7], multiple sequence alignment [1-4], profile-profile alignment [8,9], protein sequence database search [1,3], protein fold recognition [1,3,9], and protein and gene family modeling (profile HMM) [1-4].

Several powerful profile HMM tools such as HMMer [4], SAM [3], and HMMpro [2] have been developed for analyzing biological sequences. The popular tool HMMer can build a profile HMM from a family of aligned sequences (*hmmbuild*), search a profile HMM against a sequence database (*hmmsearch*), search a sequence against a profile HMM database (*hmmpfam*), and align a group of sequences against a profile HMM (*hmmalign*).

In contrast, there are only a few profile HMM visualization tools without editing functionality. HMMpro [2] can visualize HMM architecture and probabilities but, is not publicly available. HMMviewer [10] can visualize profile HMM produced by HMMer, but its visualization functionality is limited. Similarly, SAM [3] can only visualize, but only has limited editing function. Another different type of visualization tool, HMM Logo [11,12] is designed to visualize emission probabilities and transition probabilities in the popular logo style. However, HMM Logo does not provide functions to edit HMM architecture and parameters.

We also notice that some general Hidden Markov Model software includes visualization tools [13,14]. But these tools uses general input and visualization formats that are not very suitable for visualizing the special profile HMM of biological sequences.

Here we develop a visual editor for a profile HMM in the HMMer format. The HMM models produced by other tools such as SAM [3] and HHSearch [9] can be visualized after being converted into the HMMer format.

## Results

In this section, at first, we briefly introduce profile Hidden Markov Model generated by HMMer. Then we describe the HMMEditor's visualization and editing functions.

### Profile hidden Markov model

**Figure 1:**
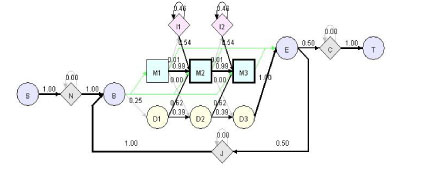
**A simple HMMer profile HMM model visualized by HHMVE**. S: start state, N: N-terminal insertion state, B: beginning state of the core of HMM, M: matching state (square), D: deletion state (yellow circle), I: insertion state (pink diamond), E: ending state of the core of HMM, J: jump state from E to B to allow domain duplication, C: C-terminal insertion state, T: terminal state. This example model has three M states (M1-3), three D states (D1-3), and two I states (I1-2). Squares (M states) and diamonds (N, I, C, J states) are emission states that can generate symbols according to emission probabilities. Circles (S, B, D, E, T) states are dummy states that do not generate symbols. The connectivity (directed edge) between two states denotes a possible transition. The number associated with an edge is the transition probability. The connectivity between B, M, D, I, E states is similar as the traditional profile HMM [1] except that B can jump to any M states and M states can jump to E. These new connectivity allows the local alignment with respect to HMM.

Profile HMM is a Hidden Markov Model representing a family of sequences [1-4]. HMMer currently uses the architecture Plan7 to support both local and global alignments between sequences and HMM (see Figure 1 for an example of profile HMM).

The whole profile HMM starts from start (S) state and ends at terminal (T) state. The core of HMM between beginning (B) and ending (E) states consists of the matching (M) states, insertion states (I), and deletion states (D). A matching state (e.g., M1-M3 in Figure 1) represents a fairly conserved position. Each matching state has a deletion state (e.g., D1-D3 in Figure 1) associated with it, allowing the deletion of the matching state (or position). Each matching state except for the last one also has an insertion state (e.g., I1-I2 in Figure 1) associated with it, allowing the insertion of additional positions after it. Unlike profile HMM in [1], transitions between I and D states are not allowed.

N and C are two special states to accommodate additional insertions before and after the conserved regions of a family of sequences, which allows local alignment between a sequence and the HMM (i.e. matching a part of a sequence against the core of HMM between state B and state E). J state joins the end of a profile HMM to the beginning. J state allows aligning a sequence against the core of a profile HMM multiple times, which is called multi-domain alignment (domain duplication). J state can model the linker (insertion) region between two domains.

Another interesting feature of the profile HMM is that there is a transition from B to each M state, and a transition from each M state directly to E state. These transitions make it possible to match only a part of the model against a sequence, allowing local alignment with respect to the HMM.

Each M, I, N, C, J states has an emission probability vector derived from input sequences. When we align a sequence against the profile HMM model, HMMer will report a log-odds score. It is the logarithm of the ratio between the probability that the sequence is generated by a profile HMM and the probability that it is generated by a null model. For the null model, the residues in a sequence are emitted according to the background distribution.

### HMMEditor

We develop a profile HMM visualization and editing tool called HMMEditor (profile Hidden Markov Model Visual Editor). HMMEditor was written in Java. Thus it works on all major operating systems (UNIX, Linux, Windows, and Mac). User can run HMMEditor in a web browser through the web start or download and install the software locally.

**Figure 2:**
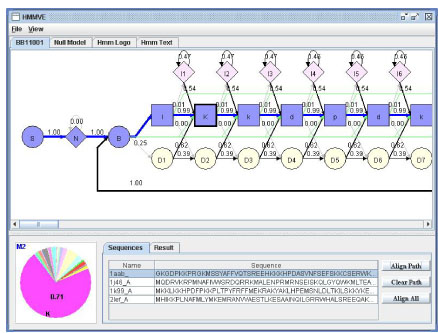
**The GUI of HMMEditor**. The top menu (File and View) allows user to open, save, and view HMM models. It also allows user to save visualized figures and sequence files. The tabs under the menu provide function to view HMM in different modes (traditional, null model, HMM Logo, and pure text). The main window under the tabs shows the visualized HMM connectivity. The numbers on the edges denote the transition probabilities. The pie chart at the bottom left shows the emission probabilities of a selected state (e.g. M2). The window at the bottom right visualizes a group of sequences. User can select each one or all them to align against the HMM.

Figure 2 shows the graphical user interface (GUI) of HMMEditor. HMMEditor has the four main features: **(1)** visualize profile HMM in different views; **(2)** edit profile HMM; **(3)** show Viterbi path; **(4)** draw HMM Logo. The features (1) and (3) are unique, which can not be found in other tools. The visualized HMM and HMM Logo can be saved into files through the GUI.

#### 1. HMM visualization

Once a profile HMM is loaded, HMMEditor is able to visualize it in the traditional layout view (Figure 2), HMM Logo view and HMM text view. It also can visualize the corresponding null model.

HMM layout view shows the structure of a profile HMM. The thickness of the transition line is proportional to the probability of the transition. The thickness of a border of an M state indicates the level of conservation. The label of each matching state denotes the most probable (consensus) residue emitted from the state.

Inside layout view, user can zoom in and zoom out the view and drag any node to get a better appearance. The layout view can be saved as a JPG or PNG file.

HMM text view shows the profile HMM in text view. The format of the text view is the same as HMMer. HMM text view is dynamically associated with the layout view. If a user edits profile HMM in the layout view, the changes will reflect in HMM text view instantly.

#### 2. HMM editing

**Figure 3:**
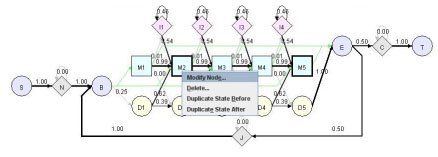
**GUI of editing HMM**.

**Figure 4:**
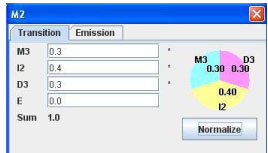
**GUI of editing transition and emission probabilities**. From the dialog, user can edit the state transition probabilities starting from state M2 (M2 -> M3, M2 -> I2, M2 -> D3, M2 -> E).

**Figure 5:**
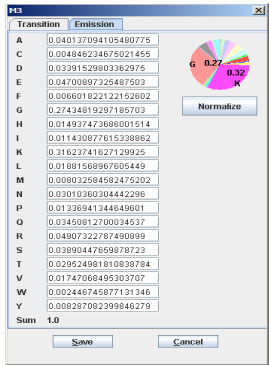
**GUI of editing emission probabilities**. This dialog is the interface of editing the emission probabilities of 20 different amino acids of state M3.

HMMer saves a profile HMM model into a text file, in which all the probabilities are converted into log-odds scores. Log-odds scores are not as intuitive as probabilities, making it hard for users to edit the model. HMMEditor provide the function to visually modify the structure and probability parameters of a profile HMM. To our best knowledge, it is the only tool equipped with this function. Figure 3 shows the interface to add, remove a state and to modify its transition and emission probabilities. User can select a state using mouse and click the right mouse button to pop up the editing menu. The delete menu lets user delete the matching state and the associated insertion and deletion states. The duplicate menu lets user to add an identical set M, I, D states before or after the current state. The modify menu allows user to modify the emission probabilities of M and I states and the transition probabilities of I, M and D states. Figures 4 and 5 show the dialogs of editing the transition and emission probabilities. Once a probability is modified, all other views will be updated immediately.

#### 3. Viterbi path visualization

**Figure 6:**
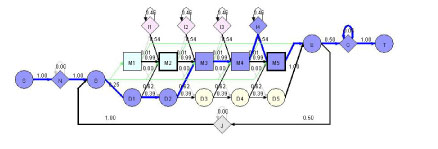
**Visualization of Viterbi path**. The thick blue line shows the Viterbi path of the sequence “MDPHE” aligned against a profile HMM consisting of 4 match states.

HMMEV provides a novel function to visualize the Viterbi (or optimal) path of aligning a sequence against a profile HMM using Viterbi algorithm. Figure 6 shows an example of aligning a short sequence “MDPHE” against a profile HMM consisting of five states. The visualization of Viterbi path help user see the conservation, deletion, and insertion of the sequence with respect the HMM of a family of sequence.

HMMEditor can read the sequence file in many popular formats such as FASTA and ClustalW [15]. Once sequences are loaded into HMMEditor, user can view the Viterbi path of each sequence. Furthermore, use can edit a sequence, i.e. add or remove residues to see how the path changes. This provides a useful means for user to adjust sequence alignments manually.

User can also align multiple sequences against a profile HMM into multiple sequence alignment and save it into a file from HMMEditor, just as *hmmalign* does.

#### 4. HMM logo

**Figure 7:**
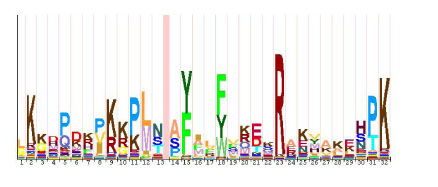
**HMM Logo**.

HMM Logo (Figure 7) [11] is a way to visualize a profile HMM, similarly as the popular motif logo used to visualize DNA binding sites [12]. Figure 7 is an HMM logo generated by HMMEditor. A HMM Logo consists of a serial of character stacks (column) separated by light red lines. Each stack represents a matching state. The lines separating neighboring stacks represent an insertion state. The height of the stack shows how significantly the emission probability of a matching state deviates from the background emission probability, i.e relative entropy (or information content). Internally, the height of each character is proportional to its information content. The width of each stack or line is determined by the hitting probability of its corresponding state. Hitting probability is the probability that a path goes through the state, which is computed efficiently using dynamic programming algorithm as in [11]. A narrow stack indicates that the state is less likely to be visited in a path. A narrow stack of a matching state means that the state is likely to be deleted instead of being visited in a path.

## Conclusions

We have developed HMMEditor, a visual editor for profile Hidden Markov Models. HMMEditor provides a convenient and appealing user interface to visualize and edit profile HMM models. It also allows user to visually adjust and align sequences against HMM. Thus, HMMEditor is a useful tool for the HMM-based biological sequence analysis in the post-genomic era. The software, source code, and web service are freely available at the HMMEditor web site [16].

## Competing interests

The authors declare that they have no competing interests.

## Authors' contributions

JD and JC designed the features of the program. JD implemented the program. JD and JC authored the manuscript. JD and JC approved the manuscript.
